# Oral Health Education in Patients with Diabetes: A Systematic Review

**DOI:** 10.3390/healthcare12090898

**Published:** 2024-04-26

**Authors:** Pinelopi Petropoulou, Ioannis Kalemikerakis, Eleni Dokoutsidou, Eleni Evangelou, Theocharis Konstantinidis, Ourania Govina

**Affiliations:** 1 Department of Nursing, University of West Attica, 12243 Athens, Greece; ppetropoulou@uniwa.gr (P.P.); ikalemik@uniwa.gr (I.K.); edokout@uniwa.gr (E.D.); elevagel@uniwa.gr (E.E.); 2 Department of Nursing, Hellenic Mediterranean University, 71410 Heraklion, Greece; harriskon@hmu.gr

**Keywords:** diabetes, oral health education, periodontitis, oral health, nursing care, telemedicine

## Abstract

Diabetes is known as a “silent epidemic” and is a public health problem that accounts for 9% of all deaths worldwide. The prevention of diabetes is a significant challenge, as its prevalence and incidence are both increasing rapidly. According to the World Health Organization (WHO), education is the cornerstone of diabetes treatment. Since the severity of oral diseases is significantly higher in diabetic patients, this systematic review aims to highlight the oral care of diabetic patients as a priority for glycemic control and the importance of education for diabetic patients’ oral health. We evaluated 20 clinical studies and 15 meta-analyses from PubMed and Google Scholar over the last five years. Their main themes are the direct relationship between diabetes and oral health, especially periodontitis, and the necessity of education and behaviors that can lead to a better quality of life. Our analysis indicated that good oral health is a critical factor of glycemic control in diabetic patients and can be enhanced by targeted educational programs, backed by long-term medical and dental follow-up. Healthcare personnel should be encouraged to develop their knowledge of oral health in relation to the disease so that behaviors can be adopted to improve patients’ quality of life. Telemedicine could also contribute to patient education and self-management of the disease.

## 1. Introduction

Diabetes mellitus (DM) is a chronic, endocrine metabolic disease with a heterogeneous and multifactorial substrate and is characterized by high blood glucose levels. It can occur either when the pancreas does not produce enough insulin, when the body cannot effectively use the insulin that it produces, or when both these conditions apply [1]. Diabetes is also called a “silent epidemic”; it is a serious public health problem and accounts for 9% of all deaths worldwide. About 90% of diabetes patients have type 2 diabetes mellitus (DM2), which causes a range of health complications, such as eye, mouth, and skin infections, cardiovascular diseases, blindness, and kidney failure, and can lead to lower limb amputation, reducing patients’ quality of life and imposing a burden on them, their families, and the wider society. It should be noted that most patients are overweight and have a high percentage of body fat, especially in the abdominal area [2].

Prevention of DM2 is a major challenge worldwide, as both its prevalence and incidence are increasing rapidly. According to the World Health Organization (WHO), by 2030, the number of diabetics will increase to at least 366 million worldwide. In Europe, the diabetic population is estimated at 66 million, a number that will rise to 89 million by 2045. However, these figures are not as dramatic as in America and many other low- and middle-income countries. The main predisposing factors for DM2 are the existence of prediabetes, obesity, age ≥ 45 years, a family history of DM2 in first-degree relatives, a lack of physical exercise, the onset of gestational diabetes or the birth of a newborn weighing > 4 kg, the presence of non-alcoholic fatty liver disease, and smoking. The main symptoms of DM2 are typical and common to all types; they include polyuria, polydipsia, polyphagia, dry mouth and/or dry skin, excessive fatigue, blurred vision, and trauma, such as cuts and bruises that are slow to heal [3].

Chronic oral diseases are significantly more common and more severe in diabetic patients. In addition to gingival infection and periodontal inflammation, there may be complications of implants, caries, dry mouth, bacterial and fungal infections, oral malodor, and slow healing of wounds from dental treatments. In addition, the Candida species are seen more often in people with diabetes than in healthy people. Periodontitis is the main and most important complication of DM, with a prevalence of 7 in 10 adults having some type of periodontal disease, and almost half of this population having moderate or severe periodontitis. Age, sex, education level, family income, smoking, oral hygiene-related behaviors, DM, and other comorbidities, such as some autoimmune diseases, have been identified as important potential risk factors for periodontitis in many studies [4].

The findings of the studies also show that DM2 is associated with an increased risk of tooth loss because of periodontal disease, possibly through the mechanism of inflammation: directly due to an inflammatory response of the gums and indirectly due to the creation of a substrate resulting from reduced saliva production secondary to medication. In particular, periodontal disease is caused by changes in collagen metabolism—and consequently, in periodontal fibers—and by the presence of microbial plaque and poor hygiene in most diabetics, resulting in resorption of the gums, the underlying connective tissue, and the jawbones, leading to loss of teeth [5].

The relation between oral health and diabetes management has long been underappreciated. The close and two-way relationship between them has only recently been proven, and it seems that the main oral diseases, such as caries and periodontitis, are related to or may exacerbate general diseases, such as diabetes. People with diabetes have a 2–3-fold greater risk for periodontitis compared to people without diabetes, but also a higher incidence of caries due to dry mouth. The progression and severity of periodontitis are also greater in people with poorly controlled diabetes. According to the American Academy of Periodontology, 50% of diabetics up to the age of 35 suffer from periodontal disease, a figure that rises to 80% at the age of 45–54 years, compared to 60% in the healthy population. A growing body of data indicates that oral inflammation has an impact on general diseases. Unfortunately, the majority of diabetic patients are unaware of the connection between diabetes and their oral health. There is a need for educational programs for diabetic patients that emphasize this relationship, explain the unpleasant effects of poor oral health on the patient’s glycemic control, and recommend behaviors that promote good oral health, but also overall wellbeing and a better quality of life.

The importance of this study is that it gathers recent evidence from surveys with a large number of samples that were conducted in different countries, and shows the direct relationship between good oral health and the control of DM. It also stresses the need to inform diabetic patients through educational programs, centered on the holistic long-term management of their oral health and their quality of life [6].

## 2. Materials and Methods

### 2.1. Study Design

We conducted a systematic review to explore the association between DM2 and periodontal diseases and to highlight oral health education as a means of improving patients’ awareness of diabetes, developing their knowledge of oral health issues, changing harmful oral health behaviors, and improving their quality of life. The study design followed the PRISMA 2020 guidelines for systematic reviews and meta-analyses.

In order to obtain a contemporary view of the topic, the last five years were taken as the reference period.

### 2.2. Search Strategy

**Figure 1:**
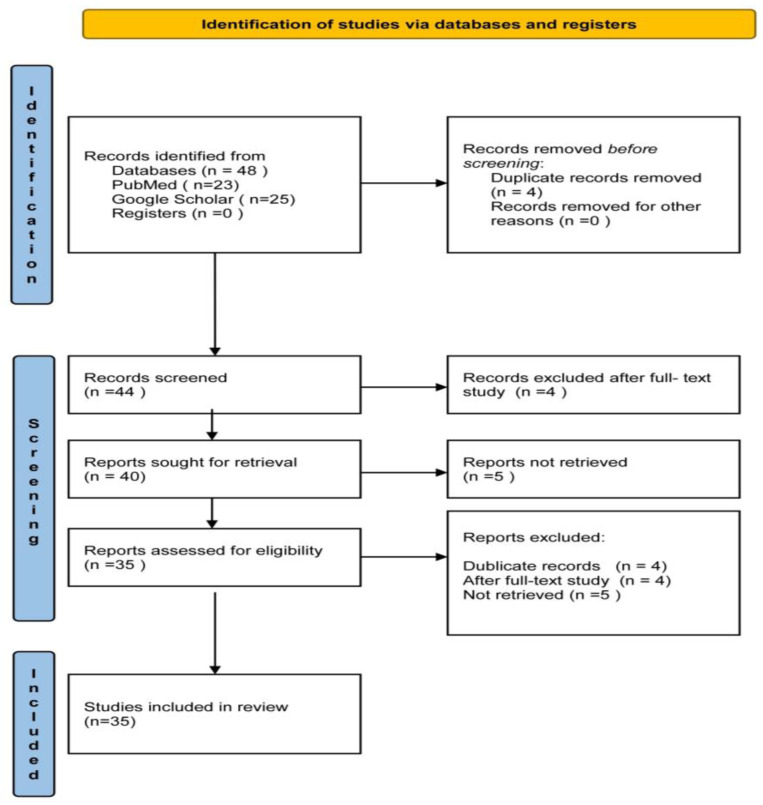
Flow chart illustrating the process of searching and locating research articles.

We searched the PubMed database for clinical trials and meta-analyses from 2019 to 2024 that provided the full research text via open access, using the Boolean search string “’oral health’ AND ‘periodontitis’ AND ‘diabetes’”. A total of 23 articles were identified, of which 3 were rejected after the full-text study; thus, 20 articles were included in the present analysis. We also searched the Google Scholar database for clinical trials in 2024 with the search string “’oral health education’ AND ’diabetes’”. From the 25 articles identified, 4 duplicate records were removed, 1 was rejected after full-text study, 5 were not retrieved, and 15 were included (Figure 1). Finally, a total of 35 articles were included in the present study: 20 clinical trials and 15 meta-analyses.

### 2.3. Selection Criteria

The inclusion criteria for this systematic review were: (1) the studies had to be clinical trials and meta-analyses; (2) from 2019 to 2024; (3) with abstract and/or full text in the English language; (4) reporting the association between DM2 and oral health; (5) reporting new therapies for periodontitis; (6) reporting oral health education and behaviors that lead to a better quality of life for diabetics.

### 2.4. Selection of Studies

The full texts of all studies that passed the first selection stage were reviewed to assess their eligibility according to the inclusion criteria. In addition, an in-depth reading of the studies was performed to obtain a list of the most decisive and defining data on patients and disease characteristics.

### 2.5. Quality Assessment and Risk of Bias

Two members of the team separately made the final choice of studies to be included. Any disagreements were discussed with a third reviewer to avoid the risk of individual bias. Finally, there was consensus in the selection of the studies.

## 3. Results

Seven cross-sectional studies, three national surveys, one qualitative study, one preliminary study, one prospective population-based study, seven randomized controlled trials, and fifteen meta-analyses, all with statistically significant *p*-values, were included in this systematic review. The US, UK, Australian, and Japanese populations were the most strongly represented, followed by patients from Germany, Peru, China, Italy, France, Finland, Brazil, Portugal, Slovenia, UAE, Taiwan, Ghana, and Iran. The main characteristics of the examined studies were recorded: the authors, the year of the study, the country, and the sample size. The studies examined the two-way relationship between periodontitis and DM2 and the simultaneous ways to control these two diseases with new methods, as well as the level of information and education provided to diabetic patients about the importance of good oral health in their disease and the quality of their lives (Table 1).

### 3.1. Two-Way Relationship between Type 2 Diabetes and Periodontitis

Periodontal disease is one of the most common chronic infections among adults, affecting more than 22% of people with diabetes. The risk of periodontitis increases with age, for active and ex-smokers, in patients with lower levels of education, and with DM, and it seems to occur in greater proportions in men than in women. It is also observed in a high percentage of the unemployed, ex-smokers, and patients with comorbidities.

The literature points to periodontitis as the main oral disease caused by impaired glycemia. Studies that examined the bidirectional relationship between diabetes and oral health appear to conclude that, during periodontal inflammation, inflammatory and microbial cells and their byproducts can cause acute production of inflammatory cytokines, interleukins, and prostaglandins, which affect insulin sensitivity or action. Inflammation has been found to be involved in the pathogenesis of DM2, promoting insulin resistance and impaired B-cell function in the pancreas. Poor glycemic control leads to the deterioration of periodontal health and the promotion of periodontal disease as a risk factor for diabetes, while diabetes increases the prevalence and severity of gingivitis and periodontitis. Developed studies have concluded that periodontal treatment can improve glycemic control by restricting inflammation, probably by improving insulin sensitivity [7,8]. Moreover, patients with DM2 and periodontitis show elevated levels of interleukin (IL)-10, while the concentration of IL-4 in diabetic subjects with periodontitis is low compared to non-diabetic patients with periodontitis, showing that there is a correlation between the level of these markers and the impairment of the immune response of patients with periodontitis and DM2. In addition, the presence of inflammatory cytokines, such as plasma C-reactive protein (CRP), links diabetes and periodontitis, as they have a direct relationship with the concentration of glycated hemoglobin (HbA1c); this shows high levels in gingivitis and periodontitis, which are significantly reduced after periodontal treatment. Furthermore, type 1 diabetes (DM1) is an autoimmune disorder characterized by a chronic inflammatory response common to periodontitis, with increased secretion of proinflammatory cytokines, including IL-1, IL-8, IL-6κ, and tumor necrosis factor-α [9]. The relationship between periodontitis and DM1 appears to be less clear than the association with DM2, and it remains to be determined whether periodontitis is a result of DM1, or whether and to what extent it contributes to the worsening of metabolic control in children and adolescents with DM1 [10]. Although the prevalence of tooth loss has decreased in recent decades, it is still a major problem of public health, since it negatively affects the individual’s quality of life. About half of the patients with diabetes have severe periodontal disease or severe gingivitis, and this eventually leads to tooth loss: studies by the American Dental Association show that 1 in 5 cases of tooth loss are related to diabetes.

### 3.2. Saliva, a Promising Biomarker Linking Oral Health to General Health

Salivary lactate levels in diabetics are higher than in healthy individuals; in advanced cases, they can reach up to five times the normal level, contributing to the formation of dental plaque and dental caries [6]. As saliva is a biofluid with over 800 identified metabolites, comparable to human serum, it can be considered as a “mirror” of oral health and used as a biological sample for the diagnosis of oral and systemic diseases by the method of vibrational spectroscopy. Saliva collection is easy, painless, and low-cost, with minimal risk of exposure to infectious agents. It is, thus, a very promising method compared to blood sampling, especially for oral cancers that are in direct contact with this biofluid [11]. Human beta defensins (hBD), especially hBD-1, are constantly present in saliva and gingival fluid and can be considered a first line of defense since they are effective against Gram-negative anaerobic pathogenic microorganisms, which are mainly responsible for periodontal infection, but also against positive bacteria. Therefore, they can also serve as indicators of existing inflammation. We should note that a promising relationship between periodontitis and hBD-1 levels is under investigation [12].

Controlling both diabetes and periodontitis can help prevent the other from occurring. Regular oral hygiene care and physical examinations are necessary for the early prevention of DM2 and periodontitis. General health and oral health are considered separately in health services; however, this mindset has begun to change in recent years, through increasing awareness of oral–systemic links and the role of oral health in improving health care [13]. Periodontal disease, for example, has been associated with an increased risk of cardiovascular disease, adverse pregnancy outcomes and complications in people with diabetes, highlighting that dental health is closely linked to overall wellbeing and quality of life. In patients with concomitant systemic diseases, such as diabetes, cardiovascular disease, chronic kidney disease, obesity and rheumatoid arthritis, from an evaluation of published scientific evidence, non-surgical periodontal therapy (NSPT) appears to be effective in combination with oral hygiene instructions. Probing with a periodontal probe and inhibition of periodontitis progression appears to lead to better periodontal outcomes in terms of the mean periodontal pocket depth, connective tissue adhesion, and bleeding [14].

### 3.3. Dental Mortality—The Role of Telemedicine

Dental mortality is a major preventable public health challenge. In more severe cases, it is life-threatening and can seriously affect a person’s quality of life. More than 800 diseases, including heart disease, stroke, diabetes, and lymphomas, as well as erectile dysfunction, are linked to diseases of the teeth and oral cavity. Early diagnosis of these diseases can lead to their effective treatment, and in many cases, save the patient’s life. Good oral hygiene is, in many cases, a key factor in the good outcome of some diseases. For example, in patients with pneumonia, improved oral hygiene and appropriate dental treatment have been found to reduce mortality rates from the disease [15]. Dental mortality disproportionately affects vulnerable and underserved populations, low-income individuals, racial and ethnic minorities, and those with limited access to healthcare. Dental pain and tooth loss can lead to discomfort, difficulty in nutritional intake and social isolation. Thus, we must emphasize the importance of preventive dentistry and regular check-ups, the importance of access to affordable dental services, education about oral health, and comprehensive healthcare policies, which must include oral health. Telemedicine, including teledentistry, is an ever-expanding practice worldwide. Teledentistry employs digital technology and telecommunications to manage dental patients and all sorts of emergencies, offering care, advice, education, and treatment. Teledentistry can manage virtual checkups to monitor treatment progress and to increase patient collaboration. Telehealth and community outreach programs are a promising strategy for eliminating gaps in access and bridging health disparities. Public health campaigns and dental education programs are instrumental in promoting oral health awareness and encouraging individuals to seek early dental care [16].

### 3.4. Recent Methods for Controlling Periodontitis and DM2

Glycated hemoglobin is a good estimate of how well blood glucose levels have been controlled over the past 2–3 months. Lifestyle and health interventions, such as exercise and physical activity, have been shown to reduce HbA1c levels and cardiovascular mortality, while improving lipid levels, blood pressure, and quality of life. In many clinical studies, periodontal health has also been shown to be significantly improved by physical activity, since it regulates multiple cytokines, especially CRP, whose high levels, like those of IL-1, are associated with periodontal inflammation [17].

At the same time, studies have shown antimicrobial photodynamic therapy (aPDT) to be an effective adjunct to standard non-surgical periodontal therapy, in the form of scaling and root planing (SRP) with 660 nm and 810 nm diode lasers. This treatment can achieve significant reduction of *Aggregatibacter actinomycetemcomitans*, *P. gingivalis*, and *T. forsythia*, essential microbes of periodontitis, sanitizing anaerobic conditions of the periodontal pockets and reducing their depth, contributing to the control of periodontal inflammation and resulting in better glycemic control in patients with DM2 and periodontitis [18]. In addition, studies have shown a reduction in HbA1c% with conservative periodontal treatment, combined with aPDT and systemic doxycycline, in non-smoking periodontal patients without severe DM2 complications. It should be noted that a 1% reduction in HbA1c appears to be equivalent to a 14% reduction in the risk of heart attack and 21% in diabetes-related deaths.

Systemic doxycycline as an adjunct to SRP in diabetic patients with periodontitis does not appear to show any additional benefit in periodontitis control over SRP alone, three months after treatment, but only in clinical gingival attachment (CAL) levels. Therefore, each clinician must weigh the pros and cons on a case-by-case basis before prescribing these antibiotics in cases of bacterial resistance since evidence shows that, in 75% of patients with chronic periodontitis, periodontal bacteria, such as *A. Actinomycetemcomitans*, are resistant to doxycycline antibiotics [19,20].

SRP improves glycemic control in DM2 patients independently of the use of adjunct metronidazole. Therefore, SRP every six months can be suggested and included as a part of overall diabetes management for patients suffering from DM2. Findings have revealed that SRP, with or without metronidazole, is significantly efficacious in not only reducing periodontitis but also achieving glycemic control in DM2 patients suffering from higher levels of HbA1c at baseline. Therefore, SRP + oral hygiene instructions without metronidazole at six months may be suggested and included in overall diabetes management [21].

Many therapeutic strategies have been developed to improve periodontal diseases in combination with conventional conservative treatment; among these, ozone (O_3_) is of particular interest. The use of ozone therapy in combination with classical periodontal treatment seems to be effective in reducing periodontal inflammation, but there is insufficient evidence, and larger studies are needed on the effectiveness of reducing the level of HbA1c in diabetic patients with periodontitis [22].

Additionally, dietary habits and deficiencies in essential nutrients, such as vitamins A, B, D, and C and calcium, have a significant impact on oral and general health that can lead to periodontal disease, angular cheilitis, mouth ulcers, dental erosion and caries, oral cancer, and breakdown of the oral mucosa. Following appropriate training, nurses, health professionals and diabetes educators have included oral health in their routine practice and can reach known vulnerable priority populations for oral health promotion. In general, the Dietary Approach to Stop Hypertension (DASH) diet and the Mediterranean diet seem to result in good oral and general health. However, more research is needed to examine dietary needs in specific groups and vulnerable populations, such as patients with DM and other chronic diseases, in relation to screening and referral for treatment, in consultation with nutritionists and dentists to promote oral and general health [23,24]. In addition, concerted efforts by professional dentists are required for oral health treatment and preventive interventions in children with special needs, since the prevalence of caries in these groups is 37%, periodontal health is at a low level, and we encounter teeth with mobility, poor gum health, high rates of plaque, and bad breath. This demonstrates a lack of knowledge and education about oral health and nutrition among students with special needs [25].

### 3.5. Importance of Oral Health Education in Patients with Diabetes

Several studies have shown that very few patients who have been diagnosed with diabetes regularly visit their dentist for periodontal examinations. Many patients are not even aware of the effect of diabetes on their oral health or that diabetes can cause the loss of their teeth [26]. According to the WHO, education is the “cornerstone” of diabetes treatment. Studies have shown that education and active surveillance are effective for controlling and treating the disease. Patients and their families need to practice a new lifestyle of monitoring blood sugar, following a proper diet, engaging in physical activity, and following preventive interventions and behaviors for appropriate oral hygiene and prevention of oral complications of DM2, based on the belief model of health. Programs aimed at training small groups to improve the quality of their oral health and control their blood sugar levels have been successfully tested, are patient-centered, and have the advantage of faster and more interesting learning, even by older patients with diabetes, compared to individual training. These programs enhance communication and collaboration between patients, raise their awareness of diabetic oral health and change negative behaviors and lifestyles. Through active monitoring in small groups, the skills related to proper oral and dental hygiene seem to be strengthened [27,28]. The symptoms of diabetes are known to affect the micro/macrovascular system and are associated with significant morbidity and mortality, and poor quality of life. Over 75% of diabetes patients live in low- or middle-income countries, where most patients receive diabetes treatment only after making private payments. The prevalence of diabetes has increased much more in low- and middle-income countries, with the largest increase observed to be 12.3% in the eastern Mediterranean region, followed by 11.1% in middle-income countries, with the lowest at 6.6% in high-income countries. Deficiencies in quality healthcare services, as well as health disparities in diabetes control and treatment, could be addressed by implementing telemedicine-based interventions, especially for remote patients. A review of the cost-effectiveness relationship of these services and their integration into national health systems is needed, with the aim of improving the epidemiological and clinical data of diabetes through the training of health personnel and community health workers [29].

Studies have found that self-care deficit assessment and supportive education programs in adult patients with diabetes changed their behavior and reduced HbA1c levels. In periods and areas with a limited health workforce, community health nurses are able to help high-risk patients with diabetes in their daily self-care to maintain their quality of life, informing patients about disease prevention, improving their self-care abilities, and helping them to change their behavior and lifestyle. This has been observed to increase awareness of diabetes as a common risk and enhance knowledge about disease prevention, even in healthy young adults [30,31].

Oral health assessment and related patient care is a largely neglected area of nursing practice, except in intensive care units and for high-risk patients. Oral hygiene interventions by nurses need to be provided to all patients, either in the hospital or in the community, as they can reveal symptoms of oral disease, manifestation of systemic diseases, side effects of drugs, or trauma. There is an urgent need for dentists to train nurses in oral hygiene care, providing them with an introduction to common oral health problems and diagnostics, the link between oral and general health, and the assessment at all stages of diabetes and patient care. Providing enhanced oral health educational materials to vulnerable populations, and those in rural and underserved areas, can improve residents’ knowledge and skill, allowing them to integrate oral healthcare into their daily practice [32,33].

Poor quality communication between patients and care providers and limited patient knowledge about the disease and its treatment are important factors associated with poor glycemic control in patients with DM2. Structured information is needed on three topics: (1) the adoption of healthy dietary measures, such as diet, weight loss, and physical exercise; (2) medication management; and (3) complications of diabetes in oral and general health [34]. According to some studies, dialogue and communication among diabetic patients, pharmacists, and community nurses about good lifestyle habits, prevention, and improved diabetes control and treatment resulted in a 0.5% drop in HbA1c in the first 6 months and 0.6% after 1 year. It appears that glycemic control can be improved by a structured and tailored information program delivered by trained community pharmacists and nurses, with better acceptance of medication and a healthier daily lifestyle. This is clinically important, since more than 40% of diabetic patients in France, as well as in other countries, do not meet their treatment goals and have HbA1c levels > 7% [19,35].

**Table 1:** Characteristics of included studies.

Authors/Year/References	Country	Type of Study	Sample Size	Health Results/*p*-Value
Karki A et al., 2023 [1]	Australia	Meta-analysis	N = 45 studies (8336 patients)	Behavior interventions in patients with DM2 for better quality of life in LMIC; *p*_1_ = 0.001, *p*_2_ = 0.060, and *p*_3_ = 0.249.
Valentim FB et al., 2021 [2]	Germany	Cross-sectional clinical study	N = 288 DM2 patients	DM2 patients’ low-level oral health and knowledge about periodontitis/diabetes relationship. Importance of integrated healthcare in this association; *p*_1_ < 0.005 and *p*_2_ < 0.05.
Wu CZ et al., 2020 [3]	West China	Meta-analysis	N = 53 studies	Bidirectional relationship between periodontitis and DM2. Severe perio-, *p* = 0.000; mild perio-, *p* = 0.007.
Ahmadinia R et al., 2022 [5]	Iran	Meta-analysis	N = 22 studies	Association between DM2 and tooth loss, *p* < 0.001.
Botelho J et al., 2019 [4]	Portugal	Cross-sectional clinical study	N = 1.064 adults	Prevalence of periodontitis/DM2 as a risk factor for periodontitis; *p* < 0.001.
Mattos MCO et al., 2019 [7]	Brazil	Meta-analysis	N = 15 studies (3894 patients)	Common inflammation biomarkers in saliva and plasma in patients with DM2 and periodontitis; *p*_1_ = 0.003 (IL-10) and *p*_2_ < 0.001 (IL-4).
Rapone B et al., 2021 [9]	Italy	Randomized controlled trial	N = 187 DM2 patients	Short-term glycemic control level and systemic inflammatory status, after non-surgical periodontal therapy in patients with diabetes and periodontitis; *p*_1_ = 0.012 and *p*_2_ = 0 (6 months).
Rapone B et al., 2020 [10]	Italy	Meta-analysis	N = 10 RCTs, each with 32 to 182 diabetic patients	Common denominator is periodontal inflammation in patients with DM2 and children and adolescents with DM1. But no results have yet emerged of a causal effect of periodontal inflammation on DM1; *p* < 0.001 and SMD = 0.36.
Yu YH et al., 2022 [36]	USA	Meta-analysis	N_1_ = 506,594 (periodontitis) N_2_ = 426,824 (DM2)	Association of periodontal disease and systemic comorbidities and bone mineral density; *p* = 0.007–0.005.
Derruau S et al., 2020 [11]	France	Meta-analysis	N = 2082, 3 oral and 7 general diseases were examined	Use of saliva for the diagnosis of oral and systemic diseases; *p* < 0.05.
Ansari Moghadam S, et al., 2024 [12]	Finland	Cross-sectional clinical study	N = 175 > 65 years	Salivary defensins HNP-1 and hBDs are less effective in the elderly against Gram-negative anaerobic pathogenic microorganisms, which are mainly responsible for periodontal infection, but also against positive bacteria; *p* = 0.019.
Poudel P et al., 2024 [13]	Australia	Meta-analysis	N = 62 studies	There is a gap in information and research around effective oral health care treatments and programs in geriatric dental care and a lack of policies and guidelines to assist both dental and medical health care professionals in integrating good oral health as part of healthy aging; *p* < 0.05.
Joseph P et al., 2023 [14]	Germany	Meta-analysis	N = 44 studies (3382 patients)	Non-surgical periodontal therapy is an effective procedure for managing periodontitis in patients with systemic diseases; *p* < 0.05.
Josy G et al., 2019 [16]	Australia	Prospective population-based study	N = 172,630 >45 years	Tooth loss and, to a lesser extent, self-rated health of teeth and gums are markers for increased risk of ischemic stroke, peripheral vascular disease, and all-cause mortality. Tooth loss is also a marker for increased risk of heart failure; *p*_trend_ < 0.05.
Rauda Al et al., 2024 [37]	UAE	Meta-analysis	N = 9 studies	Reduction of obesity through individualized health and wellness programs. Association with chronic diseases.
Wernicke K et al., 2021 [17]	Germany	Randomized controlled trial	N = 108 DM2 patients	Physical activity is an oral health-promoting measure in patients with DM2 and physical activity significantly reduces HbA1c concentrations; *p*_1_ = 0.005 (BOP) and *p*_2_ = 0.010 (HbA1c).
Brinar S et al., 2023 [18]	Slovenia	Randomized controlled trial	N = 24 patients with DM2 and periodontitis	Antimicrobial photodynamic therapy (aPDT) by diode laser is an effective adjunct to standard non-surgical periodontal therapy, contributing to the control of periodontal inflammation; *p* < 0.05.
Yap KCH et al., 2019 [19]	Malaysia	Meta-analysis	N = 6 studies	Systemic doxycycline as an adjunct to scaling and root planing does not significantly improve CAL for periodontal status or reduction of HbA1c levels in the treatment of diabetic patients with periodontitis; *p* < 0.05.
Cao R. et al., 2019 [20]	China	Meta-analysis	N = 14 RCTs, 629 patients	NSPT treatment with aPDT + Doxy has the best efficacy in periodontal treatment and lowering HbA1c% of non-smoking CP without severe DM2 complications. Further studies are needed to confirm the results.
Rapone B.et al., 2023 [22]	Italy	Randomized controlled trial	N = 100 patients with DM2 and periodontitis	Gaseous ozone therapy as supporting treatment for periodontitis in patients with DM2 has beneficial effect on HbA1c. Larger studies are needed to confirm the results; *p*_1_ = 0.007 and *p*_2_ = 0.001.
Patterson-Norrie T, et al., 2024 [23]	Australia	National Survey clinical research	N = 149 dieticians	Dietary needs in specific groups and vulnerable populations, such as patients with chronic diseases, in relation to screening and referral for treatment to promote oral and general health; *p* = 0.001–0.028.
Altun E et al., 2021 [24]	Germany	Cross-sectional clinical study	N = 6209 patients with severe and none/mild periodontitis	Association between diet habits and initiation/progression of periodontitis; *p* < 0.001.
Amuasi AA et al., 2024 [25]	Ghana	Cross-sectional clinical study	N = 121 participants from two Special Schools	Poor oral health in children with special needs, decays and gums bleeding/DMFT value of 2.82.
Mohseni Homagarani Y, et al., 2023 [27]	Iran	Meta-analysis	N = 11 studies, each with 88 to 350 diabetic patients	Oral health has an essential role in diabetic patients’ quality of life; SMD = 0.148.
Hsu YJ et al., 2021 [28]	Taiwan	Randomized controlled trial	N = 76 (n_1_ = 39, n_2_ = 37)	NSPT periodontal therapy with the Community Health Workers (CHW) strategy improved short-term clinical treatment outcomes (PPD and CAL), reduced HbA1c high levels, and exerted a positive effect on long-term OHQoL in DM2 patients; *p* < 0.05.
Hartono V et al., 2024 [29]	Indonesia	Randomized controlled trial	N = 40 patients with periodontitis	Implementation of interventions based on telemedicine can improve the cognitive and clinical data of patients with periodontal disease; *p* < 0.05.
Hargraves VM, et al., 2024 [31]	USA	Qualitative clinical research	N = 44 patients (n1 = 21, n2 = 23)	Access to oral health care is limited for patients residing in assisted living facilities. There is a need to inform policymakers and advocates about access to oral health care as they develop new approaches.
Hernández-Vásquez A, et al., 2020 [32]	Peru	Cross-sectional clinical study	N = 41,330 children age (1–11 years)	Need to increase use of oral health services in rural settings in order to reduce existing rural–urban inequalities and access to information on adequate oral hygiene practices; *p* < 0.001.
Yanagita T et al., 2023 [34]	Japan	Survey research clinical study	N = 28 universities	Results from universities that conducted ‘‘pharmacology role-play’’. Pharmacology role-play is effective in (1) understanding medical treatment; (2) understanding patients’ feelings; (3) improvement of mental attitude and motivation as health professionals; (4) positive influence on study attitude.
Hayashi T et al., 2022 [35]	Japan	Survey research clinical study	N_1_ = 176 hospitals with dental care, N_2_ = 321 hospitals cooperating with dentists	Society requires the involvement of dental hygienists in the field of palliative care to provide good oral care to patients with terminal illnesses and contribute to improving their quality of life.
Chou Κ. et al., 2019 [19]	Malaysia	Meta-analysis	N = 6 RCTs, each with 15 to 35 diabetic patients	Systemic doxycycline as an adjunct to scaling and root planing does not significantly improve CAL for periodontal status or reduction of HbA1c levels in patients with diabetes; SMD = 0.13.
Cai Y et al., 2024 [38]	South China	Cross-sectional clinical study	N = 678 college students	College students neglect oral hygiene and have limited understanding of their own oral issues. Considering the current poor oral health status observed in other age groups, such as children and older adults, it is strongly recommended to promote oral health education and provide guidance to all populations; *p* < 0.001 (to 8 factors).
Qureshi A et al., 2021 [21]	Ojha University Campus, Karachi, Pakistan	Randomized controlled trial	N = 150 DM2 patients with moderate and severe periodontitis	NSPT with and without metronidazole is significantly efficacious, not only for reducing periodontitis but also for glycemic control in DM2 patients with periodontitis suffering from higher levels of HbA1c at baseline; *p*_1_ < 0.020 (3 months) and *p*_2_ < 0.06 (6 months).
Toda K et al., 2019 [39]	Tokyo, Japan	Preliminary clinical study	N = 21 DM2 patients	Interventions and behaviors of the diabetic patients, which with education and follow-up can prevent, slow down, and control DM2 and its unpleasant consequences; *p* < 0.0167.
Shungin D et al., 2019 [40]	UK	Meta-analysis	N = 33 studies ~500,000 people	This investigation used detailed clinical measures in combination with genetically validated proxy phenotypes to investigate the two major dental diseases, caries and periodontitis; *p* < 0.05.

## 4. Discussion

This systematic review analyzed and synthesized 35 studies that highlight the seriousness of diabetes as a chronic disease and the seriousness of its complications expressed in different body systems, especially the oral cavity. The findings reveal that preventive measures for DM2 should aim to modify the environmental factors responsible for the disease, in interaction with the genetic factors of its appearance [38].

Specifically for the oral cavity, the close relationship between DM2 and periodontal disease was delineated, their pathophysiology and their two-way relationship were analyzed, and the modern approaches to their treatment and control were examined. The control of periodontal inflammation with conservative treatment and oral hygiene guidelines, as well as whatever other modern aids may be available—such as the simultaneous administration of low photodynamic therapy, ozone therapy, exercise and proper nutrition, medical and dental re-examination every six months—are critical interventions in the daily lives of diabetic patients, aimed at controlling their disease. The simultaneous administration of doxycycline or metronidazole antibiotics must be judged on a case-by-case basis by the attending physician in accordance with other parameters, given the possibility of microbial resistance [31,39]. It has been shown that periodontal inflammation is involved in the pathogenesis of DM2 and leads to poor glycemic control, burdening the health and quality of life of diabetics. A growing number of recent studies are examining saliva as a promising biomarker for detecting elements of dental and systemic diseases. It has been estimated that dental caries in permanent teeth and periodontitis were the leading and 11th most prevalent cause of disease worldwide in 2016. It has been reported that the genetic contribution and heritability of caries and periodontitis is as high as 50%. Increased understanding of genetic factors may inform etiological theory and clinical management [40]. Periodontal disease has been also associated with DM2 and osteoporosis, but the underlying genetic mechanisms for these associations remain unknown. Research to explore a potentially shared biology between periodontitis, DM2, and bone mineral density has identified 14 candidate genetic association loci shared between DM2 and periodontal disease, but more future research is needed to explore how the young sites are related to the pathophysiology of periodontitis and comorbidities [36].

The provision of correctly structured information to patients with DM2 about the disease and its effects on the body, their education on an appropriate lifestyle, and the daily management of the disease in the long term are contemporary public health challenges worldwide, given the increase in morbidity and mortality of the disease and the chronic financial burden on the patient, family, and community. Proper education from a young age in school about healthy lifestyles and the promotion of oral health through information and new telemedicine technologies can lead to a generation that can control disease and overall wellbeing. On a larger scale, to promote general and oral health and disease prevention nationwide, health literacy should be integrated into the curriculum for primary school children aged 6–12 so that they can use this knowledge to improve their health and their general wellbeing. Creating a healthier and more informed generation depends on children who can make health decisions and build lifelong healthy habits and lifestyles that are able to navigate modern health care systems for a healthier life [37]. Health staff and community nurses can play a leading role in this direction, including oral hygiene education and early diagnosis for referral in chronic oral health-related conditions, in daily nursing practice. There seems to be a relative lack of studies on the education of nursing staff, and how it can enable them to instruct patients with DM2, and/or other immunosuppressed patients with systemic diseases and poor oral health, about proper oral hygiene, to impart knowledge of the impact of their disease on their general and oral health, and to how their quality of life may be improved. Therefore, there is a need for more studies that will strengthen and verify our results in terms of their general applicability.

The limitations of our study include the search of clinical studies in only two databases for a limited time in the last five years, in only English, and according to the study selection criteria mentioned in the methodology chapter.

## 5. Conclusions

In this study, we present a good number of studies with a large number of patients, from which we can draw the conclusion that the good oral health of diabetic patients, especially the control of periodontitis with classical and modern methods, is a determining factor of glycemic control, and vice versa. We also describe methods and behaviors, such as smoking cessation, obesity reduction, dietary habits, and physical exercise, that simultaneously contribute to the control of DM2 and oral health and the improvement of the patients’ quality of life. We conclude that the long-term control of diabetes and oral health can be achieved with systematic medical and dental check-ups every six months, and with the contribution of telemedicine for remote and infirm patients.

Unfortunately, however, patients with diabetes seem to have insufficient knowledge about oral health and its connection with their disease, and as a result, they do not seek and do not have access to appropriate preventive oral health services or an appropriate lifestyle. For this reason, public health campaigns and dental education programs by appropriate health personnel are vital for the management of diabetic patients, as well as patients with other comorbidities. This review could also guide healthcare professionals to more easily recognize high-risk patients and be a critical resource for healthcare providers. It would particularly benefit those seeking to integrate oral health education into the management of patients with diabetes, especially DM2. It is necessary to elucidate the close relationship between oral and systemic health in diabetic patients through integrated oral health policies based on the education of patient and healthcare staff, prioritizing prevention and health promotion in order to save lives and improve the quality of life and well-being of diabetic patients and their community. Further studies on the changing needs of DM2 patients and the development of new technologies will certainly contribute to these goals.

## Data Availability

Not applicable.
